# Comparative Analysis of Protocadherin-11 X-Linked Expression among Postnatal Rodents, Non-Human Primates, and Songbirds Suggests Its Possible Involvement in Brain Evolution

**DOI:** 10.1371/journal.pone.0058840

**Published:** 2013-03-18

**Authors:** Eiji Matsunaga, Sanae Nambu, Mariko Oka, Kazuo Okanoya, Atsushi Iriki

**Affiliations:** 1 Laboratory for Symbolic Cognitive Development, RIKEN Brain Science Institute, Wako, Japan; 2 Department of Life Sciences, Graduate School of Arts and Sciences, University of Tokyo, Tokyo, Japan; 3 ERATO Okanoya Emotional Information Project, JST-ERATO, Saitama, Japan; 4 Emotional Information Joint Research Laboratory, RIKEN Brain Science Institute, Wako, Japan; Utrecht University, The Netherlands

## Abstract

**Background:**

Protocadherin-11 is a cell adhesion molecule of the cadherin superfamily. Since, only in humans, its paralog is found on the Y chromosome, it is expected that protocadherin-11X/Y plays some role in human brain evolution or sex differences. Recently, a genetic mutation of protocadherin-11X/Y was reported to be associated with a language development disorder. Here, we compared the expression of protocadherin-11 X-linked in developing postnatal brains of mouse (rodent) and common marmoset (non-human primate) to explore its possible involvement in mammalian brain evolution. We also investigated its expression in the Bengalese finch (songbird) to explore a possible function in animal vocalization and human language faculties.

**Methodology/Principal Findings:**

Protocadherin-11 X-linked was strongly expressed in the cerebral cortex, hippocampus, amygdala and brainstem. Comparative analysis between mice and marmosets revealed that in certain areas of marmoset brain, the expression was clearly enriched. In Bengalese finches, protocadherin-11 X-linked was expressed not only in nuclei of regions of the vocal production pathway and the tracheosyringeal hypoglossal nucleus, but also in areas homologous to the mammalian amygdala and hippocampus. In both marmosets and Bengalese finches, its expression in pallial vocal control areas was developmentally regulated, and no clear expression was seen in the dorsal striatum, indicating a similarity between songbirds and non-human primates.

**Conclusions/Significance:**

Our results suggest that the enriched expression of protocadherin-11 X-linked is involved in primate brain evolution and that some similarity exists between songbirds and primates regarding the neural basis for vocalization.

## Introduction

Protocdherin-11 X-linked (Pcdh11X) is a cell adhesion molecule belonging to the cadherin superfamily [1]–[2]. The cadherin superfamily is composed by classic cadherins, desmosomal cadherins, Flamingo/Celsr, desmosomal cadherins, T-cadherin, 7D-cadherins, CDH15 & 23, Fat & Dachsous, Flamingo/Celsr, calsyntenins, Ret and protocadherins [3]–[7]. Protocadherins are further subdivided into two subgroups: clustered (Pcdh-alpha, -beta, -gamma) and non-clustered protocadherins (delta protocadherins). Pcdh11X belongs to delta protocadherins. It is known that various molecules belonging to the cadherin superfamily play multiple roles in axon guidance, dendritic spine formation, specific synapse formation and regulation of neuronal activity [3]–[7]. Since only humans have two Pcdh11 genes on the X and Y chromosome (Pcdh11X and Pcdh11Y, respectively), it is expected that the molecule plays some role in human brain evolution, or determination of sex differences [8], [9]. In fact, it has been recently reported that a child with small chromosome deletions in both Pcdh11X and Pcdh11Y genes showed developmental language delay, and had severe defects in language production and understanding [10]. Additionally, intellectual disability was identified in 2 brothers with a gene duplication in intron 2 of Pcdh11X [11]. Although the dysfunction of Pcdh11X/Y in human psychiatric disorders has been proposed, and immunohistochemical analysis of Pcdh11X expression has been examined in postmortem human brains [12], invasive human studies are strictly limited; therefore, appropriate animal models are necessary to allow the exploration of molecular and behavioral functions of Pcdh11X.

The common marmoset (*Callithrix jacchus*) is a small, New World primate, endemic to the forests of Brazil. Marmosets have several noteworthy characteristics, such as group living, parental care, monogamous pair bonds, a variety of vocal communications [13], and a capacity for cognitive learning [14]. Since it is easy to handle and breed marmosets in the laboratory, marmosets have been commonly utilized for neuroanatomical, physiological, and ecological studies. Furthermore, transgenic technology has been recently applied to this animal, and transgenic marmosets with germ-line transmission have been established [15]. Moreover, analysis of immediate early gene expression suggests that marmosets posses a neocortical network for vocalization similar to that of humans [16], [17]. Although only human beings are able to use the language on the earth, animals have a part of language faculty such as vocalization or sequential learning, therefore, various animals have been used to explore the neural basis of language faculty [18]. Thus, by the virtue of their phylogenetic proximity to humans compared to other animals such as mice, marmosets are considered as ideal model animals for studying the molecular mechanisms of human cognitive functions or primordial structure of human vocal pathways.

However, other model animals have commonly been used for studying the faculty of language. Songbirds such as finches learn their vocalizations during the juvenile stage, as do human babies. Even though they are phylogenetically far from humans, it has been recently demonstrated that anatomical features and the expression of certain genes in songbirds are analogous to humans [19]–[21]. Therefore, these species are thought to be excellent model animals for the learning of syntax and vocal learning.

Here, we performed *in situ* hybridization analyses of Pcdh11X expression in developing common marmoset, mouse, and Bengalese finch brains, to reveal spatial and temporal expression patterns during the various developmental stages of non-human primates, rodents, and songbirds. Firstly, by comparing developing postnatal mouse and marmoset brains, we examined the possible involvement of Pcdh11X in mammalian brain evolution. Secondly, by examining developing postnatal songbird brains, we examined the possible involvement of Pcdh11X in animal vocalization, or the faculty of human language.

## Materials and Methods

### Ethics Statement

All research protocols were approved by the Animal Care and Use Committee of RIKEN (#H22-2-216 for marmosets and mice, #H18-2B002 for Bengalese finches), and conformed to the National Institutes of Health (NIH) Guidelines. All surgery was performed under deeply anesthetized conditions, and all efforts were made to minimize suffering.

### Animals and sample preparation

Common marmosets (*Callithrix jacchus*) were purchased from the research resource center of the RIKEN institute. Until just before experiments, all juvenile marmosets were housed with their parents and adult marmosets were housed individually in a breeding room with other breeding cages. The animals were kept at 27°C with 50% humidity on a 12-h light–dark cycle. We used 1 P0 male, 1 P2 male, 1 P28 male, 1 P56 male, 1 P85 male, and 2 adult (more than 1.5 years old) female marmosets. Mice were purchased from Nihon SLC Inc. (Shizuoka, Japan), and bred in our laboratory facilities. We used 4 P0, 1 P12, 3 P14, and 4 postnatal mice that were 8 weeks old. Marmosets and mice were deeply anesthetized by an intramuscular injection of sodium pentobarbital (75 mg/kg or 150 mg/kg, respectively, Dainihon Seiyaku, Osaka, Japan) and then perfused with phosphate buffered saline (PBS; pH 7.4) solution. After dissection, their brains were embedded in a Tissue-Tek optimal cutting temperature compound (Sakura Fine Technical Co. Ltd. Tokyo, Japan), and frozen with dry ice in preparation for cryosectioning. Frozen sections for either *in situ* hybridization, or thionine staining for neuroanatomical references, were cut serially at 16 or 20 µm thicknesses using a cryostat (Leica Microsystems Inc., Wetzlar, Germany). We used the same series of brain sections of male Bengalese finches we had made previously (n = 4, each stage) (Birds were sacrificed after they were deeply anesthetized by an intramuscular injection of sodium pentobarbital (50 mg/kg)) [22]. Brain tissues were dissected and placed in Qiazol Lysis reagent (QIAGEN, Hilden, Germany) to extract the total RNA, which was purified using an RNeasy Lipid Tissue Mini Kit (QIAGEN).

### Isolation and cloning of cDNA

The marmoset Pcdh11X cDNA fragments (Genbank accession number, AB693123) were isolated from the neonatal brain of a female marmoset using a reverse transcription-polymerase chain reaction. The Bengalese finch Pcdh11X cDNA fragments (AB745409) were isolated from adult male cDNA as described previously [22]. ‘The primers used to isolate marmoset and Bengalese finch cDNA were 5′-AAGTGGAGGTTGCCATTTTG-3′, 5′-CTGCAGTCTCCAGGAGGAAC-3′ and 5′-GCAACCTGCTCAAGGATCTC-3′, 5′-AAAGCACTGTCCCATTCACC-3′, respectively. The primer sequences were based on human or zebra finch cDNA sequences. The probes used corresponded to the extracellular domain of human Pcdh11X cDNA, which is included in all human Pcdh11X transcription variants (variant a–h), and Pcdh11Y transcription variants (variants a–c). The cDNA fragments were inserted into pGEM-T Easy cloning vectors (Promega, Madison, WI). The plasmids for the marmoset or Bengalese finch Pcdh11X antisense or sense probes were linearized using SpeI or NcoI to release the fragment, and the probes were synthesized using T7 or SP6 RNA polymerase (Roche Diagnostics, Rotkreuz, Switzerland), with a digoxigenin (DIG)-labeling mix (Roche Diagnostics). The mouse Pcdh11X clone (Genbank accession No. AK039071) was obtained from the FANTOM full-length cDNA project [23]. We cut the cDNA with NotI or BamHI, for the antisense or sense probes respectively, to release the fragment, and the probes were synthesized using T3 or T7 RNA polymerase (Roche Diagnostics).

### *In situ* hybridization of tissue sections

*In situ* hybridization of all tissue sections was performed using the same method [22]. The sections were post-fixed for 10 min in PBS with 4% paraformaldehyde solution, and then washed 3 times in PBS for 3 min. The slides were delipidated with acetone, acetylated, and washed in PBS with 1% Triton-X100 (Wako Pure Chemical, Osaka, Japan). Slides were then incubated at room temperature with a hybridization buffer containing 50% formamide (Wako Pure Chemicals), 5 × standard sodium citrate (SSC), 5× Denhardt's solution (Sigma, St. Louis, MO, USA), 250 µg/mL yeast tRNA (Roche Diagnostics), and 500 µg/mL DNA (Roche Diagnostics). The sections were then hybridized at 72°C overnight in the hybridization buffer with RNA probes. They were then rinsed in 0.2× SSC for 2 h, and blocked for 2 h in a solution of 0.1 M Tris (pH 7.5) and 0.15 M NaCl with 10% sheep serum. The slides were next incubated overnight with an alkaline phosphatase (AP)-conjugated anti-DIG antibody (Roche Diagnostics). After washing, AP activity was detected by adding 337.5 mg/mL of nitroblue tetrazolium chloride, and 175 mg/mL of 5-bromo-4-chloro-3-indolyl phosphate (Roche Diagnostics). All images were captured with a NanoZoomer 2.0 (Hamamatsu photonics, Hamamatsu, Japan). Photoshop software (ver. CS5; Adobe Systems, Mountain View, CA, USA) was used to change the color images to black-and-white, crop unnecessary areas, and juxtapose panels, and to enhance the contrast and brightness, if required. We used the terminology determined by Reiner et al., Franklin & Paxinos, and Paxinos et al., [24]–[26].

## Results

**Figure 1:**
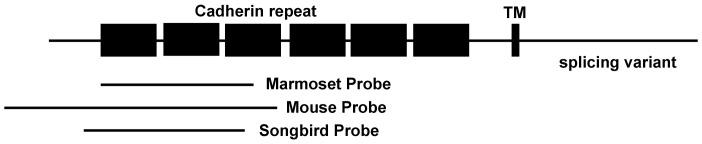
Schematic representation of Pcdh11X probes used in this study. All *in situ* hybridization probes were made from cDNAs encoding the extracellular domain of Pcdh11X.

In this study, we used probes encoding the N-terminal region of Pcdh11X, which corresponded to the region where all human Pcdh11X transcripts are included (Fig. 1). We detected strong signals with the antisense probe, but no clear staining was seen with the sense probe. Expression patterns are summarized in Table1 and 2.

**Table 1:** Summary of Pcdh11X expression in developing postnatal marmoset and mouse brain.

		Marmoset		Mouse	
		juvenile (P28)	adult	juvenile (P14)	adult
**Neocortex**					
I					
II		*	*	***	***
III		*	*	***	*
IV		***	*	*	
V		*	*	*	*
VI		***	***	***	***
**Basal ganglia**					
lateral septal nucleus	LS	*	*	***	***
medial septal nucleus	MS	*	*	***	***
nucleus accumbens	Acb				
diagonal band of Broca	DB	*	*	***	***
ventral pallidum	VP	*	*	***	*
caudate nucleus	Cd	*		*	*
putamen	Pu	*		*	*
globus pallidus	GP	*	*	***	***
claustrum	Cl	*	*		
**Amygdala**					
amygdalohippocampal area	AHi	***	***	***	***
basolateral amygdaloid n.	BL				
basomedial amygdaloid n.	BM	***	***	***	***
bed n. of stria terminalis	ST	*	*	***	***
central amygdaloid n.	Ce	*	*	***	***
corticaloid amygdaloid n.	Co	***	***	***	***
lateral amygdaloid n.	La				
medial amygdaloid n.	Me	*	*	***	***
**Hippocampal area**					
ammon's horn	CA1			*	
	CA3	***	***	*	*
dentate gyrus	DG			*	
presubiculum	PrS	***	***	***	***
subiculum	S				
entorhinal cortex	Ent	***	***	***	***
piriform cortex	Pir	***	***	***	***
**Epithalamus**					
medial habenular nucleus	MHb	***	***	*	*
lateral habenular nucleus	LHb	***	***	***	***
**Thalamus**					
Anterior thalamic group					
anterodorsal thalamic n.	AD				
anteromedial thalamic n.	AM				
anteroventral thalamic n.	AV				
Lateral thalamic group					
ventral anterior thalamic n.	VA	*	*	***	*
ventral lateral thalamic n.	VL				
ventromedial thalamic n.	VM	***	*	***	***
ventral posterolateral thalamic n.	VPL			*	
ventral posteromedial thalamic n.	VPM			*	
lateral dorsal thalamic n.	LD	*		*	*
Medial thalamic group					
central lateral thalamic n.	CL				
paracentral thalamic n.	PC				
central medial thalamic n.	CM	*	*	*	*
centromedian thalamic n.	CMn	***	*	-	-
parafacicular thalamic n.	PaF	***	*	***	***
mediodorsal thalamic n.	MD				
paraventricular thalamic n.	PV	*		*	
reuniens thalamic n.	Re				
Posterior thalamic group					
lateral geniculate n.	LG	*		*	
medial geniculate n.	MG				
lateral posterior n.	LP	*		*	*
posterior n.	Po	*		*	
pulvinar	Pul	*		-	-
**Ventral thalamus**					
reticular thalamic n.	Rt			***	*
subthalamic n.	STh	*	*	***	***
zona incerta	ZI	*	*	***	***
**Hypothalamus**					
anterior hypothalamic n.	AH	*	*	*	*
arcurate hypothalamic n.	Arc	***	*	*	***
dorsomedial hypothalamic n.	DM	*	*	*	*
lateral hypothalamic area	LH	*	*	*	*
mammilary body	MB	*	*	*	*
medial preoptic area	MPA	*	*	*	*
paraventricular hypothalamic n.	Pa	*	*	*	*
posterior hypothalamic area	PH	*	*	*	*
ventromedial hypothalamic n.	VMH	***	***	***	***
**Brainstem**					
substantia nigra pars compacta	SNc	*	*	***	***
substantia nigra pars reticulata	SNr	*	*	***	***
periaqueductal gray	PAG	*	*	***	***
pontine reticular nucleus	Pn	***	***	***	***
nucleus ambiguus	Amb	***	***	***	***
superior colliculus	SC	***	***	***	***
inferior colliculus	IC	*		*	
nucleus of lateral lemniscus	LL	*	*	*	
superior olive	SO			*	
Cranial nerve	nIII	*	*	***	***
	nIV	*	*	***	***
	nV	***	***	***	***
	nVI	***	***	***	***
	nVII	***	***	***	***
	nVIII			***	***
	nX	***	***	***	***
	nXII	***	***	***	***
inferior olive	IO	***	***	***	***

No homologous areas to CMn and Pul are found in mouse brain (-). High expression ***, moderate expression *.

**Table 2:** Summary of Pcdh11X expression in the developing postnatal Bengalese finch brain.

		Bengalese finch			
		P30	P60	adult	Corresponding area
**Vocal control areas**					
HVC	HVC	***	***		VLPFC
robust nucleus of the arcopallium	RA	***	***		Motor Cortex
dorsal medial nucleus of the midbrain	DM	***	***	***	PAG
tracheosyringeal hypoglossal nucleus	nXIIts	***	***	***	Amb
lateral magnocellular nucleus of the anterior nidopallium	LMAN				VLPFC or ACC
Area X	AreaX				CPu
dorsal lateral nucleus of the thalamus	DLM				VA/VL
**Auditory areas**					
caudal medial nidopallium	NCM	***	***	***	Auditory Cortex
caudal mesopallium	CM	***	***	***	Auditory Cortex
nucleus ovoidalis	Ov				MGN
lateral mesencephalic nucleus	MLd				IC
intermediate lateral lemniscal nucleus	LLi				LLi
superior olivary nucleus	SO				SO
cochlear nuclei	CN	***	***	***	CN
**Other areas**					
nucleus taenia	TnA	***	***	*	Amygdala
hippocampus	Hp	***	***	***	Hp
habenula	Hb	***	***	*	Hb

High expression ***, moderate expression *.

### Pcdh11X expression in the developing postnatal marmoset brain

*In situ* hybridization analysis revealed that Pcdh11X exhibited strong/dense expression in a few restricted brain areas where its function may play essential roles, and moderate/weak expression in various brain areas where its function may be less important. We will only describe here notable areas of expression.

#### Pcdh11X expressions in the cerebral cortex

**Figure 2:**
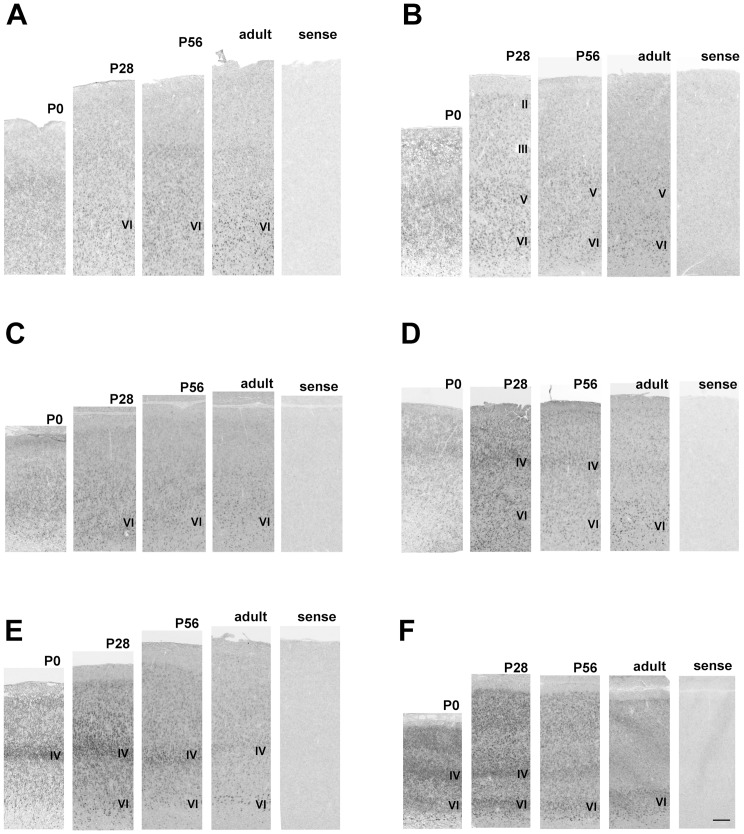
Pcdh11X expression patterns in the cerebral cortex of the marmoset brain. Ventrolateral prefrontal cortex (VLPFC) (A), motor cortex (B), anterior cingulate cortex (ACC) (C), auditory cortex (D), parietal cortex (area TE) (E), and visual cortex (F). No staining is seen with the sense probe. Strong expression is seen in layers IV and VI. Expression in layer IV is decreased during development. Scale bars represent 250 µm.

The cerebral cortex of the neonatal marmoset brain has a clear stratified structure, suggesting that cortical development of the neonatal marmoset is more progressed than the neonatal mouse, as has been previously mentioned [27]–[29]. Pcdh11X expression was seen throughout the cerebral cortex, including the cingulate and retrosplenial cortices, from the neonatal to the adult stage. Pcdh11X expression was detected in layers II–VI of the cerebral cortex (Fig. 2) at the neonatal stage. The expression level was generally reduced during development. Some areal differences were also seen. For example, no clearly stratified expression pattern was seen at the neonatal stage in either the prefrontal or the auditory cortex, and expression became localized within deeper layers during development (Fig. 2A–D). In contrast, strong Pcdh11X expression was seen in layer IV in the temporal area TE at the neonatal stage (Fig. 2E). Strong Pcdh11X expression was seen in layers IV and VI of the visual cortex at the neonatal stage (Fig. 2F). Expression in the upper cortical layers was reduced, as seen in the other areas, and clear expression was only seen in the VI layer at the adult stage.

#### Pcdh11X expression in the cortico-striatal-thalamic pathway

**Figure 3:**
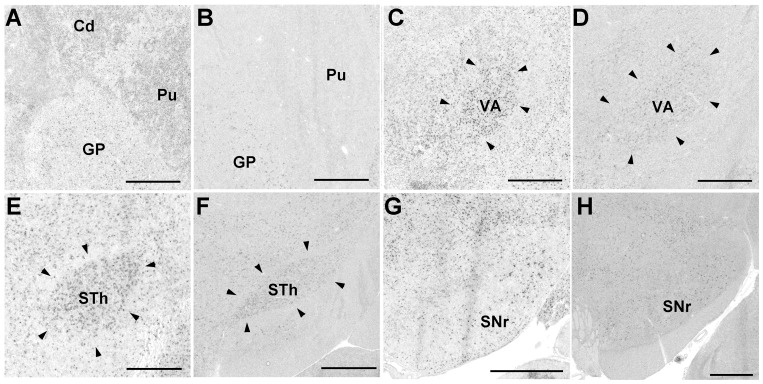
Pcdh11X expression in the cortico-striatal pathway of the marmoset brain. GP at P0 (A) and adult stage (B); VA at P2 (C), and adult stage (D); STh at P2 (E), and adult stage (F); SNr at P2 (G), and adult stage (H). Pcdh11X is weakly expressed in Cd and Pu at P0, but their expressions have disappeared by the adult stage. Scale bars represent 1 mm.

Pcdh11X expression was evident in the brain areas corresponding to the cortico-basal ganglia motor pathway. Although Pcdh11X expression in the caudate nucleus (Cd) and putamen (Pu) was only evident in the early postnatal stage, and expression in these regions was reduced during development (Fig. 3A, B), moderate Pcdh11X expression was seen in other areas of the brain, such as the globus pallidus (GP), ventral anterior thalamus (VA), subthalamic nucleus (STh), and the substantia nigra reticular part (SNr), throughout the postnatal stage (Fig. 3C–H).

#### Pcdh11X expression in the amygdala

**Figure 4:**
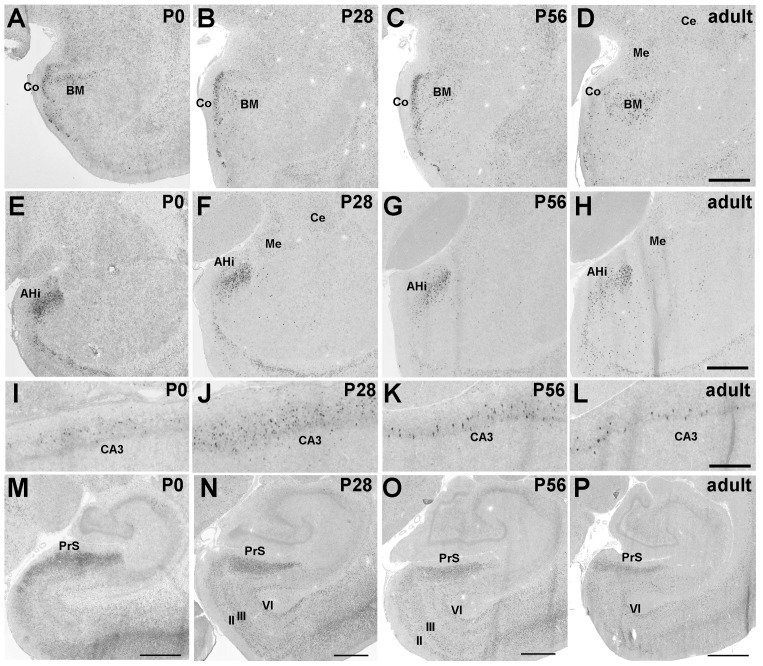
Strong Pcdh11X expression is seen in brain areas involved in memory and emotion. Pcdh11X expressions in BM (A–D) and AHi (E–H), in the CA3 region of the hippocampus (I–L), and the PrS (M–P) in the developing postnatal marmoset brain. Scale bars represent 500 µm (L), 1 mm (D, H), and 2 mm (M–P).

Strong Pcdh11X expression was seen in the amygdalo-hippocampal transition area (AHi), basomedial amygdaloid nucleus (BM), and cortical amygdaloid area (Co) (Fig. 4A–H). Moderate expression was also seen in the central amygdaloid nucleus (Ce), medial amygdaloid nucleus (Me), and the bed nucleus of the stria terminalis (ST) (Table 1). These expression patterns were maintained up to the adult stage.

#### Pcdh11X expression in hippocampal areas

Clear Pcdh11X expression was only seen in the CA3 region (Fig. 4I–P) of the hippocampus. Interestingly, Pcdh11X-expressing cells were sparsely distributed through the pyramidal layer of CA3, suggesting that Pcdh11X exhibited neuronal subset specific expression (Fig. 4I–L). Strong expression was also found in the presubiculum (PrS), entorhinal cortex (Ent), (Fig. 4M–P), and piriform cortex (Pir) (Table 1). Pcdh11X expression was seen in layers II, III, and VI of the Ent. This expression in the upper layer was reduced during development, and clear expression was only seen in layer VI at the adult stage (Fig. 4M–P). Dense Pcdh11X expression was seen in the upper layer of PrS, and this was maintained up to the adult stage (Fig. 4M–P).

#### Pcdh11X expression in the brainstem

**Figure 5:**
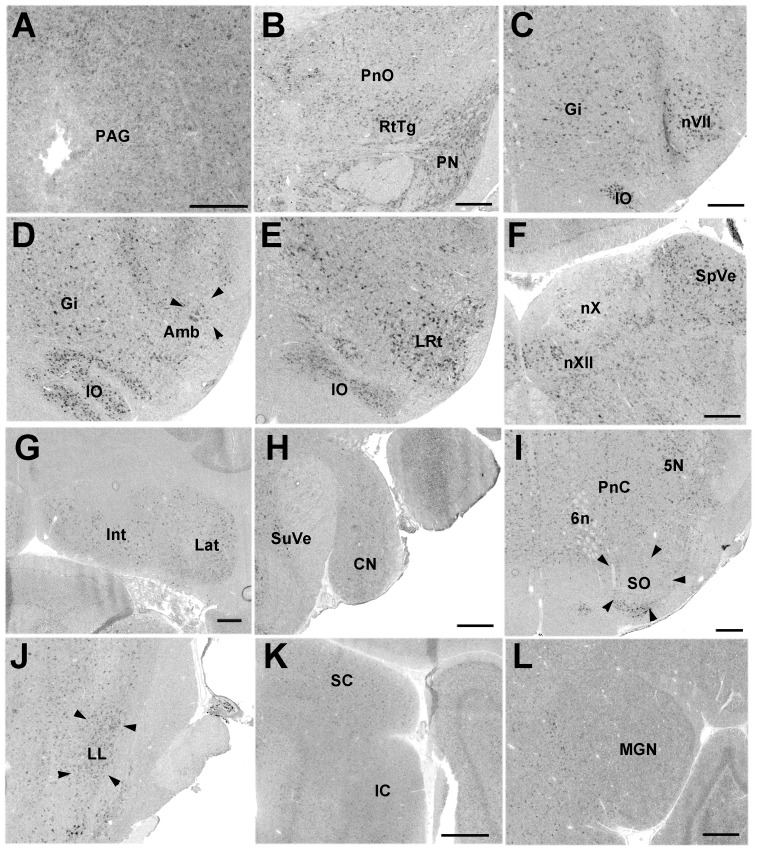
Pcdh11X expression in the brainstem. PAG (A) in P56 marmoset brain. Pontine reticular formation (B), nVII (C), Amb (D), lateral reticular nucleus (LRt) (E), and nXII (F), cerebellar nuclei (G), CN (H), SO (I), LL (J), IC (K), and medial geniculate nucleus (MGN) (L) in adult marmoset brain. Gi, gigantocellular reticular nucleus; Int, interposed cerebellar nucleus; IO, inferior olive; Lat, lateral cerebellar nucleus; PN, pontine nucleus; PnC, pontine reticular formation, caudal part; Rt, reticular thalamic nucleus; RtTg, reticulotegmental nucleus; SpVe, spinal vestibular nucleus; SuVe, superior vestibular nucleus. Scale bars represent 500 µm.

Pcdh11X expression was detected in the nuclei of cranial nerves III–VII, IX, X, and XI (we did not have a slice including nXI). Pcdh11X was expressed in vocally related areas, such as the periaqueductal gray (PAG), pontine reticular nucleus oral part (PnO), nucleus ambiguus (Amb), and nXII (Fig. 5A–F). In contrast, weak, or no expression was found in auditory areas such as the cochlear nuclei (CN), superior olivary nucleus (SO), lateral lemniscal nucleus (LL), and the inferior colliculus (IC) (Fig. 5H–K). Pcdh11X expression was not detected in the thalamic auditory area (Fig. 5L).

#### Pcdh11X expression in other areas

**Figure 6:**
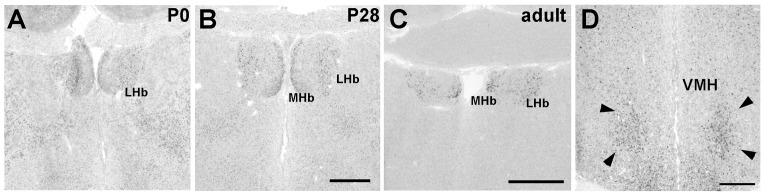
Pcdh11X expression in the habenula (A–C) and hypothalamic region (D). VMH at the adult stage (D). Scale bars represent 1 mm.

Strong Pcdh11X expression was evident in the medial and lateral habenula (corresponding to MHb and LHb) (Fig. 6A–C). In the hypothalamus, a broad but weak expression was found, and strong expression was only detected in the ventromedial hypothalamus (VMH) (Fig. 6D).

### Pcdh11X expression patterns in the mouse brain

We next performed *in situ* hybridization of Pcdh11X using another mammalian species, i.e., the mouse, in order to examine whether the expression pattern in the marmoset brain was conserved in other mammalian species. We found clear Pcdh11X staining with the antisense probe, as has been previously reported in mice [1], [2], [30], [31] and rats [32], [33], but no clear staining was seen with the sense probe.

**Figure 7:**
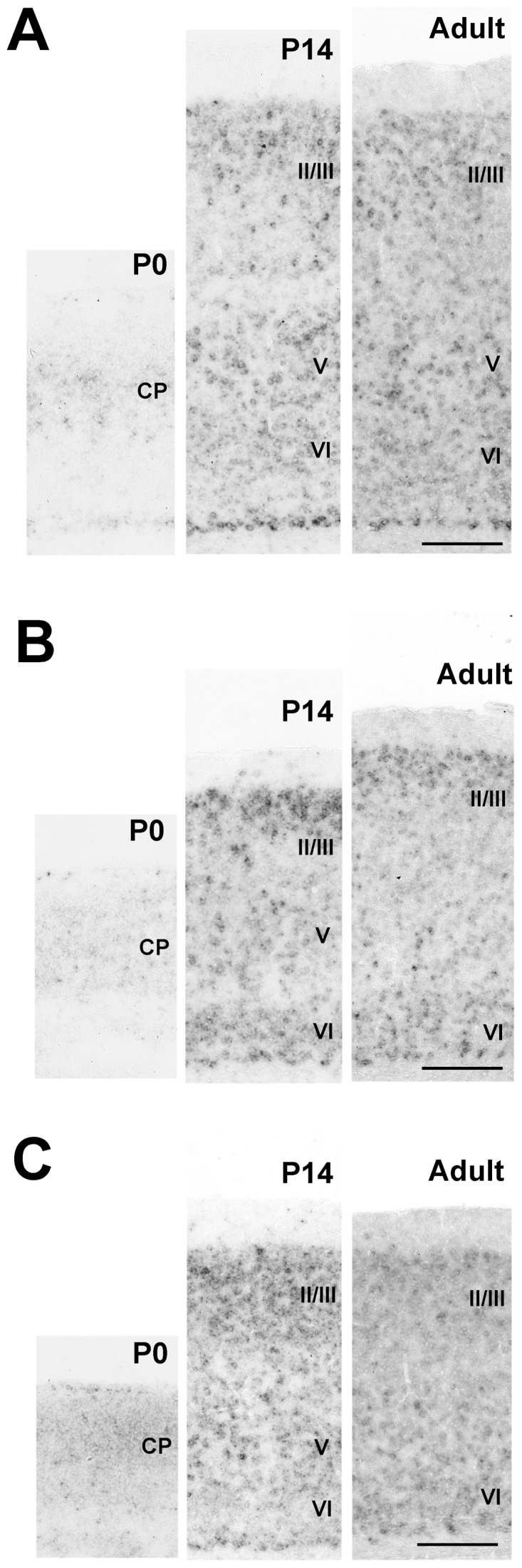
Pcdh11X expression in the developing postnatal cerebral cortex of the mouse brain. Frontal cortex (A), auditory cortex (B), and visual cortex (C). In contrast to the marmoset brain, strong Pcdh11X expression is seen in the upper layer, while no clear layer IV expression is seen. Scale bars represent 200 µm.

**Figure 8:**
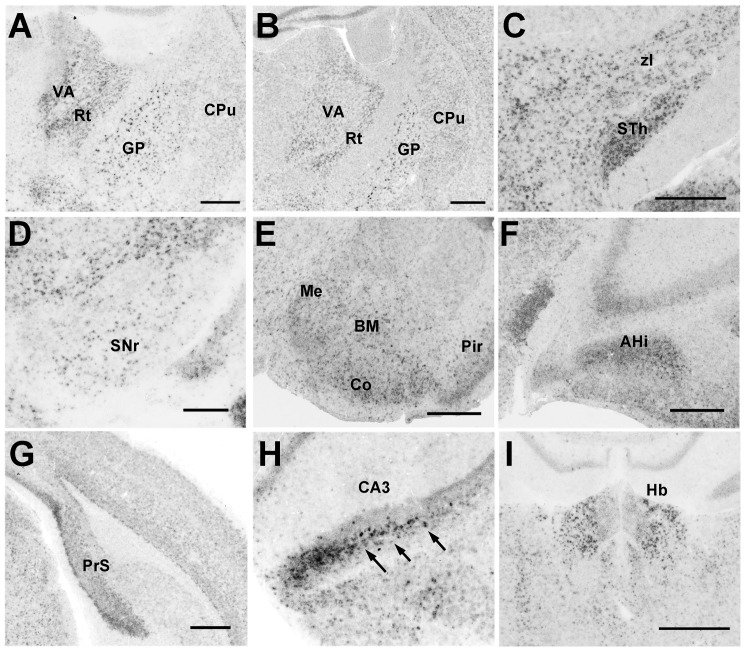
Pcdh11X expression in the adult mouse brain. Caudate putamen (CPu) at P14 (A) and the adult stage (B). STh (C), SNr (D), amygdala (E), AHi (F), PrS (G), CA3 (H), and habenula (I). Scale bars represent 500 µm. Arrows indicate strongly Pcdh11X-expressing cells (H).

**Figure 9:**
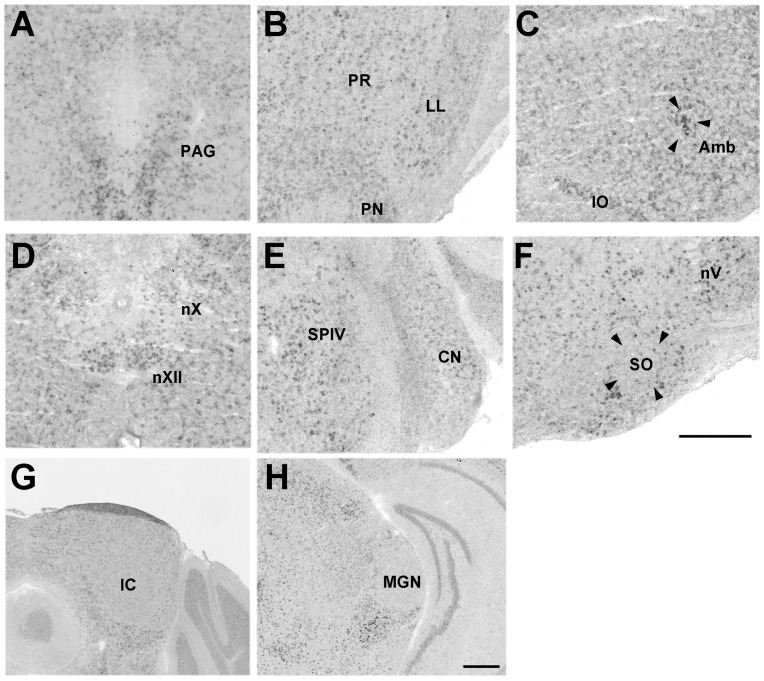
Pcdh11X expression in vocal and auditory pathways of the brainstem and the thalamic nucleus of adult mouse brain. PAG (A), LL (B), Amb (C), nXII (D), CN (E), SO (F), IC (G) and medial geniculate nucleus (MGN) (H). PR, prerubral field; SPIV, spinal vestibular nucleus. Scale bars represent 500 µm.

The expression patterns of Pcdh11X are broadly similar for mice and marmosets. Strong Pcdh11X expression was seen in the cerebral cortex (Fig. 7), cortico-basal ganglia-thalamic loop (Fig. 8A–D), amygdala (Fig. 8E, F), PrS, and CA3 in the hippocampal areas (Fig. 8G, H), habenula (Fig. 8I), and the vocal nuclei in the brainstem (Fig. 9A–D); little clear expression was seen in the brainstem auditory areas (Fig. 9E–G) and thalamic auditory area (Fig. 9H). However, precise expression patterns were different from the marmoset in some brain areas. Even though Pcdh11X expression was seen in the cerebral cortex, as with marmosets, the layer specific expression pattern appeared to be different (Fig. 7). Pcdh11X was strongly expressed in layers II–III, but no clear layer IV expression was seen in the mouse brain. In contrast to marmosets, Pcdh11X expression in the upper cortical layers was retained at the adult stage. Pcdh11X expression was also maintained in the mouse caudate nucleus (Figs. 3B, 8B). Only the lateral region of the mouse habenula strongly expressed Pcdh11X (Figs. 6A–C, 8I). With these exceptions, the expression patterns of Pcdh11X were generally similar for mice and marmosets.

### Pcdh11X expression in the songbird brain

Next, we examined Pcdh11X expression in a vocal learning bird, Bengalese finch. In the songbird brain, there is a neural network called ‘song system’ dedicated to vocal learning and production [19]–[21]. The song system develops postnatally, and in accordance with the development, birds acquire their songs through sensorimotor learning. During the vocal learning process, birds first listen to the father's song (sensory learning stage), then practice singing by themselves (sensorimotor learning stage), and finally copy the father's song. To explore the possible involvement of Pcdh11X in vocal learning, we analyzed Pcdh11X expression in P30 (during the sensory learning stage), and P60 (during the sensorimotor learning stage), and more than 120 days (after the crystallized stage, adult stage).

#### Pcdh11X expression in the vocal system

**Figure 10:**
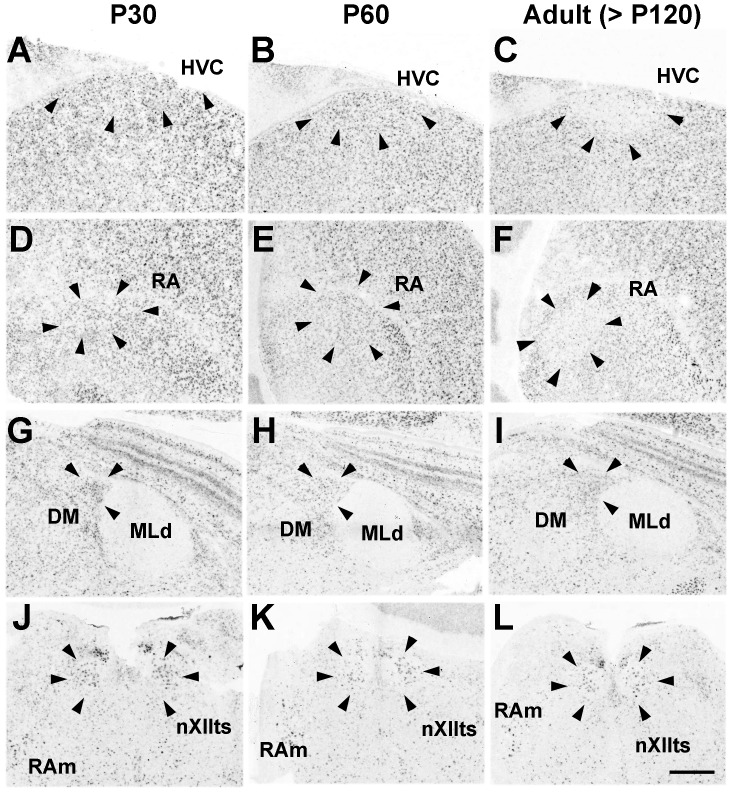
Pcdh11X expression in the vocal system of songbirds. Pcdh11X is expressed in vocal production areas such as HVC (A–C), RA (D–F), DM (G–I), and nXIIts and RAm (J–L). Pcdh11X expression in the telencephalic nuclei is reduced during development (C, F). In contrast, no Pcdh11X expression is seen in the auditory area, MLd (G–I). Scale bars represent 500 µm.

**Figure 11:**
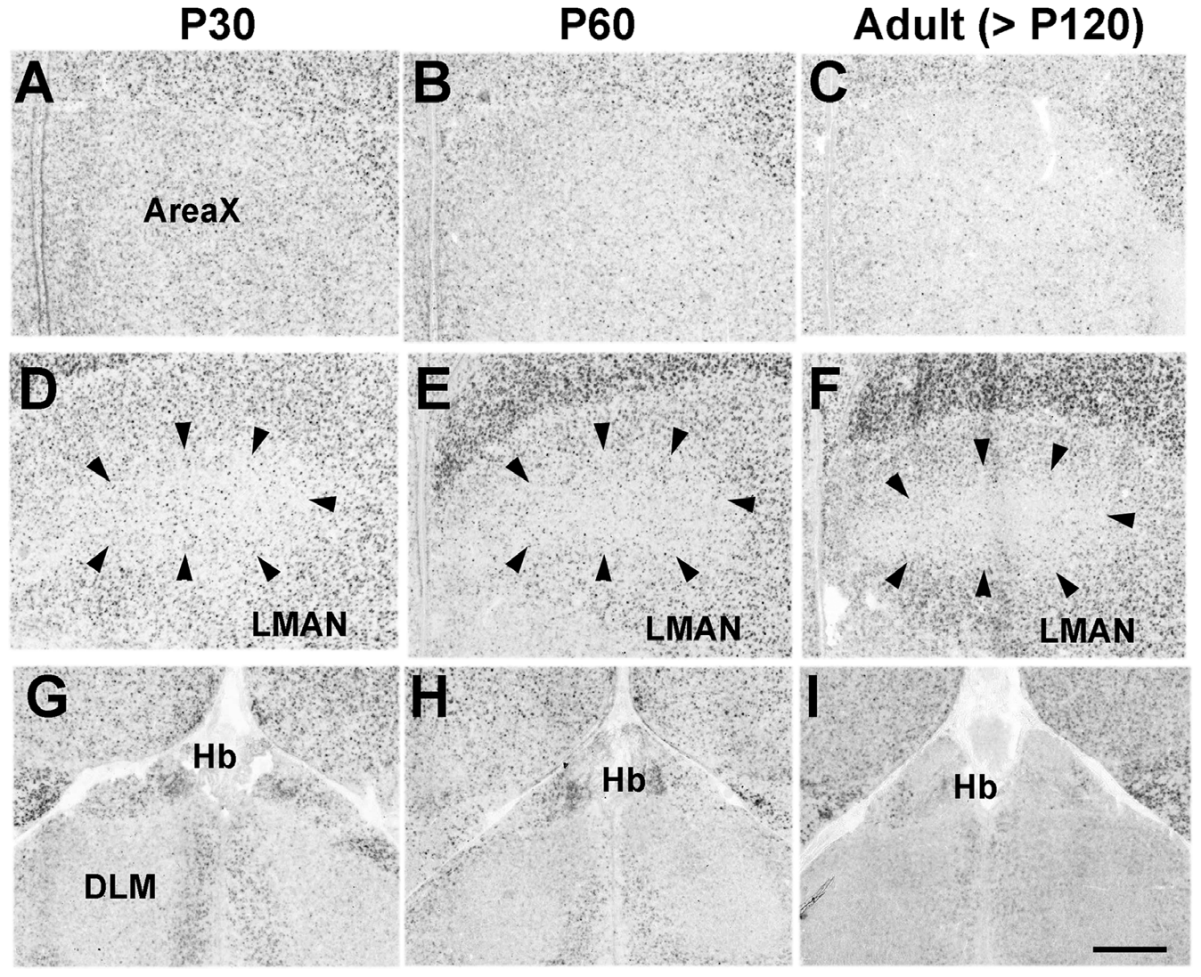
No clear Pcdh11X expression was seen in the basal ganglia loop of the vocal learning pathway. Area X (A–C), LMAN (D–F), and DLM (G–I). Scale bar represents 500 µm.

As with marmosets and mice, Pcdh11X was broadly expressed in the nuclei associated with vocal production, such as the HVC, robust nucleus of arcopallium (RA), dorsal medial nucleus of the midbrain (DM), tracheosyringeal hypoglossal nucleus (nXIIts), and nucleus retroambiguus (RAm) (Fig. 10). Interestingly, Pcdh11X expression in the telencephalic vocal nuclei, HVC and RA, was decreased during the sensorimotor learning stage (Fig. 10A–F), although expression in the surrounding non-vocal areas in the nidopallium and arcopallium was maintained. On the other hand, Pcdh11X was not expressed within nuclei of the cortico-striatal pathway such as Area X, the dorsal lateral nucleus of the thalamus (DLM), and the lateral magnocellular nucleus of the anterior nidopallium (LAMN) (Fig. 11).

#### Pcdh11X expression in the auditory system

**Figure 12:**
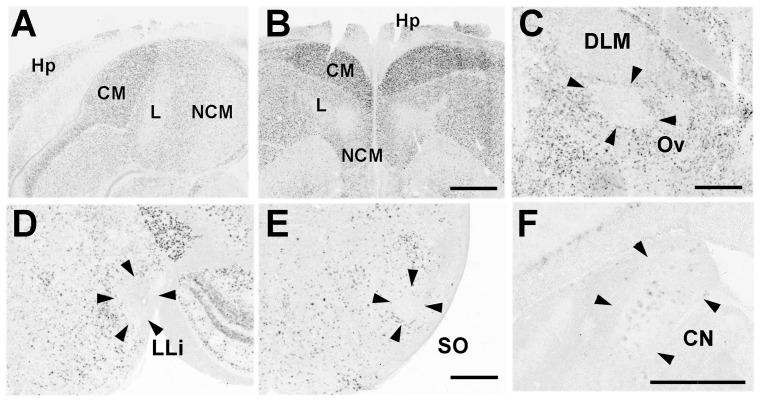
Pcdh11X expression in the auditory pathway of songbirds. Telencephalic auditory areas in P30 and adult birds (A, B). Clear Pcdh11X expression is seen in CM and NCM. As in marmosets and mice, no clear expression is seen in the auditory pathway outside the telencephalon (C, D, E), except for CN (F). L, Field L. Scale bars represent 1 mm (B) and 500 µm (C, E, F).

Since vocal learning is auditory-dependent, we examined Pcdh11X expression in the auditory system. Pcdh11X expression was detected in telencephalic auditory areas such as the caudal medial nidopallium (NCM) and the caudal mesopallium (CM) in both juvenile and adult stages (Fig. 12A,B), whereas weak, or no clear expression was seen in auditory areas outside the telencephalon, such as the nucleus ovoidalis (Ov) (Fig. 12C), lateral mesencephalic nucleus (MLd) (Fig. 10G–I), intermediate lateral lemniscal nucleus (LLi) and SO, except for the CN (Fig. 12D–F).

#### Pcdh11X expression in other areas

**Figure 13:**
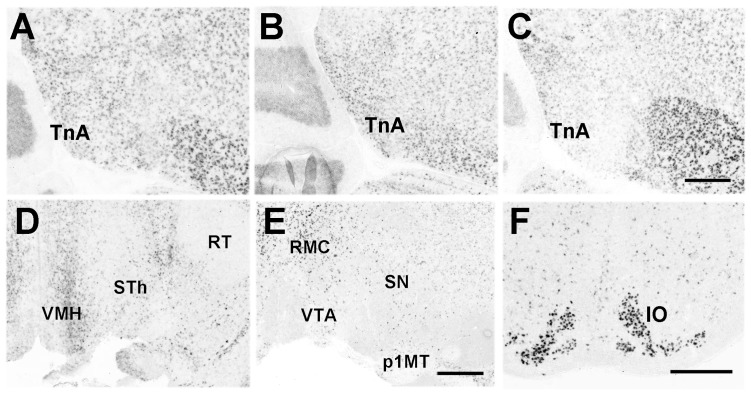
Pcdh11X expression in other brain areas of songbirds. Pcdh11X expression in the area homologous to the amygdala at P30 (A), P60 (B), and adult (C). VMH (D), SN (E), and IO (F) of adult birds. p1MT, p1 medial terminal nucleus; RMC, reticular magnocellular nucleus; RT, nucleus rotundus; VTA, ventral tegmentum area. Scale bars represent 500 µm.

Pcdh11X was also expressed in other brain areas. As with both marmosets and mice, Pcdh11X was expressed in the functionally homologous area of the amygdala (taenia nucleus [TnA]) (Fig. 13A–C), in hippocampal areas (Fig. 12A, B), Hb (Fig. 11G–I), VMH (Fig. 13D), substantia nigra (SN) (Fig. 13E), and IO (Fig. 13F).

## Discussion

**Figure 14:**
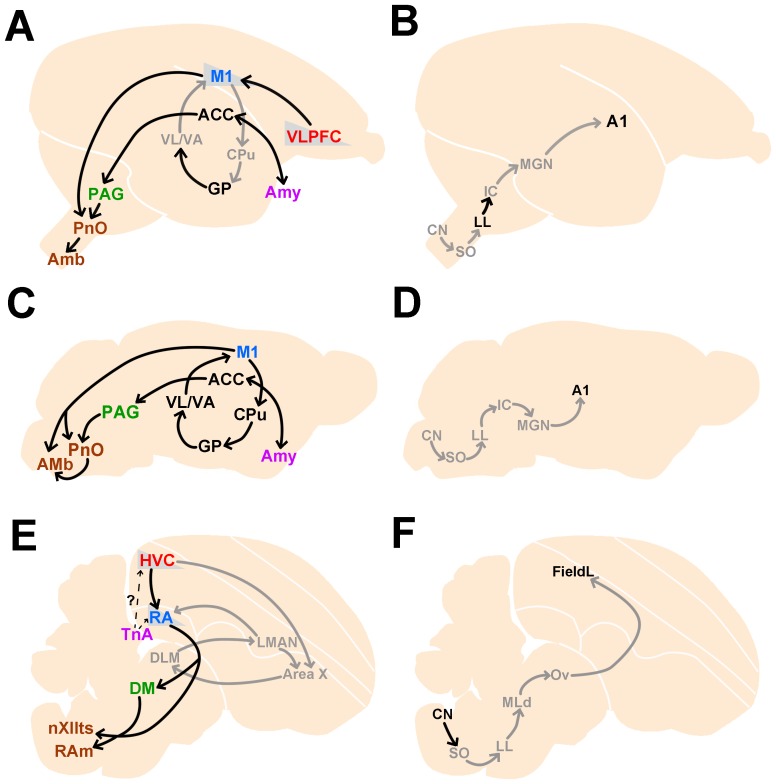
Summary of Pcdh11X expressions in vocalization-related areas (A, C, E) and auditory-related areas (B, D, F) of marmosets (A, B), mice (C, D) and Bengalese finches (E, F). Each color code indicates analogous brain area between avian and mammalian species. Gray color indicates areas with no clear Pcdh11X expressions. It has been proposed that mouse has a direct projection from the telencephalic motor area to vocal control nucleus in the brainstem, as songbirds [91]. A1. Auditory area 1; Amy, amygdala.

Although Pcdh11X expression patterns have been analyzed in some rodents and humans, this is the first account published to date regarding expression in non-human primates and songbirds. In this study, we examined Pcdh11X expression in the developing postnatal marmoset brain, and found dense expression in some restricted brain areas including the amygdala, hippocampus, habenula, and nuclei of the brainstem, with some differences between marmosets and mice. Furthermore, we examined Pcdh11X expression in a songbird, the Bengalese finch, and found some similarity between expression patterns in songbirds and non-human primates, even though they have different structure of the telencephalon. We summarized homologous and analogous Pcdh11X expressions of marmosets, mice and Bengalese finches in Fig. 14.

### Comparison of Pcdh11X expression patterns between rodents, non-human primates, and humans

Overall, Pcdh11X expression in mice and marmosets was similar. However, there were some notable differences between the two. Firstly, Pcdh11X exhibited different layer specific expression patterns in the cerebral cortex between species. Furthermore, Pcdh11X showed a different layered expression pattern in different cortical areas of the marmoset. Since the cerebral cortex is the area that has most dramatically expanded during primate evolution, changes in the expression pattern of Pcdh11X might lead to the acquisition of primate-specific changes in terms of neural connections and functions, or areal differences. Examination of the postmortem human brain revealed that Pcdh11X is expressed broadly, with no layer specific expression in the prefrontal cortex [12]. Thus, Pcdh11X expression may differ even between different primate cortices. Secondly, outside the cerebral cortex, the expression levels appeared to be different in some brain areas, even though the spatial expression patterns were similar between the 2 species. In contrast to the broad expression found in mice, strong expression was restricted to some limited areas of the marmoset brain, such as AHi, BM, MHb, and PrS. These subtle differences might affect brain functions. With regard to the human brain, there are limited data available on Pcdh11X expression patterns [12], [34]–[38]. Further detailed studies in postmortem human brains, or other primate species, are necessary to further examine this possibility.

### Similar and convergent Pcdh11X expression between songbirds and non-human primates

Even though birds and mammals are far separated in their evolutionary lineage, many similarities have been identified between vocal learning in birds and humans, on the molecular level as well as the behavioral level [19]–[21]. In the present study, we found similar Pcdh11X expression patterns in vocal areas between marmosets and Bengalese finches. In both species, strong Pcdh11X expression was detected in vocal production pathways (HVC, RA, DM, nXIIts, and RAm in songbirds, and VLPFC, AAC, PnO, and Amb in marmosets), but its expression was absent in other parts of the cortico-striatal pathway (Area X, DLM, and LMAN in songbirds, and caudate putamen and thalamic motor nuclei in marmosets). Interestingly, Pcdh11X expression in the upper layer of the marmoset prefrontal and motor cortex, and in the telencephalic song nuclei HVC and RA, was clear only in the juvenile stage, and declined by adulthood. It has been proposed that the formation and maturation of HVC-RA connection is essential for vocal development of songbirds. In both species, such a transient Pcdh11X expression may be involved in the formation and maturation of the vocal pathway during development, or in neural plasticity.

In mammalian species, direct or indirect connections from the amygdala to the PAG may be involved in emotional behaviors, including vocalization. Although the areas of the bird brain developmentally homologous to the mammalian amygdala remain to be elucidated, the taenia nucleus is thought to be the anatomically and functionally homologous [39]. Pcdh11X expression was also detected in this area, suggesting the involvement of Pcdh11X in emotion-dependent vocalizations by controlling neural connections.

Thus, in both avians and mammals, Pcdh11X expression has some commonality in the vocal production pathway.

### Possible involvement of cadherin superfamily in brain evolution

Previously, many papers have been published about extensive gene expression analysis of classic cadherins and protocadherins [22], [30]–[33], [40]–[63] and their functional roles in brain morphogenesis, neural connections, neural activity or behaviors [64]–[83]. Cadherin expressions are developmentally regulated and their altered expressions are suggested to be involved in brain morphogenesis and functions [22], [64], [65], [77]–[79], [84]–[86]. Species differences in gene expressions were also demonstrated [54]–[56], [59], [60], and the possible involvement of cadherins in brain evolution and diversity have been proposed.

In contrast to classic cadherins, protocadherins possess different features. Protocadherins show weak homophilic interactions [87] and their cytoplasmic domains are diverse among their family members [5], suggesting their roles more than just cell adhesion molecules. Although protocadherins themselves show weak homophilic interactions [87], they show strong homophilic cell-cell adhesions through the interaction to classic cadherins [88]. Moreover, regulatory regions of some protocadherins are modified by DNA methylation in relation to early life experience or mental pathogenesis [89], [90], suggesting the epigenetic control of their expressions. Thus, compared to classic cadherins, it appears that protocadherins play more diverse and more complicated roles in brain development and functions via diverse interactions with other cadherins, various downstream signaling pathways and their epigenetic controls.

Our previous study in Bengalese finches revealed that type-II classic cadherin expressions were changed from Cdh7-positive to Cdh6-positive in the RA nucleus during the development [22], and that lentiviral Cdh7 overexpression in the RA nucleus blocked vocal development [78]. In vitro analysis using hippocampal culture neurons showed that Cdh6 and Cdh7 showed distinct properties in spine formation and neuronal activity [79]. In vocal nuclei such as HVC and RA, other type-II classic cadherins such as Cdh9, Cdh10 and Cdh12 showed song nuclei-related expressions [22]. Thus, combinatorial expression of Pcdh11X with other cadherins and their developmental changes may coordinate and diversify neural connections or vocal plasticity. Various type-II cadherins also express differentially in the developing marmoset cortex (EM et al., unpublished data). Such mechanisms may work in primate vocalization or learning systems. Precise gene expression analysis of various cadherins and protocadherins, and functional analysis of these molecules in mice, marmosets and songbirds using electroporation, viral vectors or RNA interference should be performed to discover Pcdh11X functions in brain evolution of various cognitive functions or the use of language.
